# Supplementary figures and images for: An unconventional regulatory circuitry involving Aurora B controls anaphase onset and error-free chromosome segregation in trypanosomes
Source: bioRxiv. 2024 Jan 20:2024.01.20.576407. Preprint. [Version 1] doi: 10.1101/2024.01.20.576407 (PMC10827227; doi:10.1101/2024.01.20.576407)

Figure S1

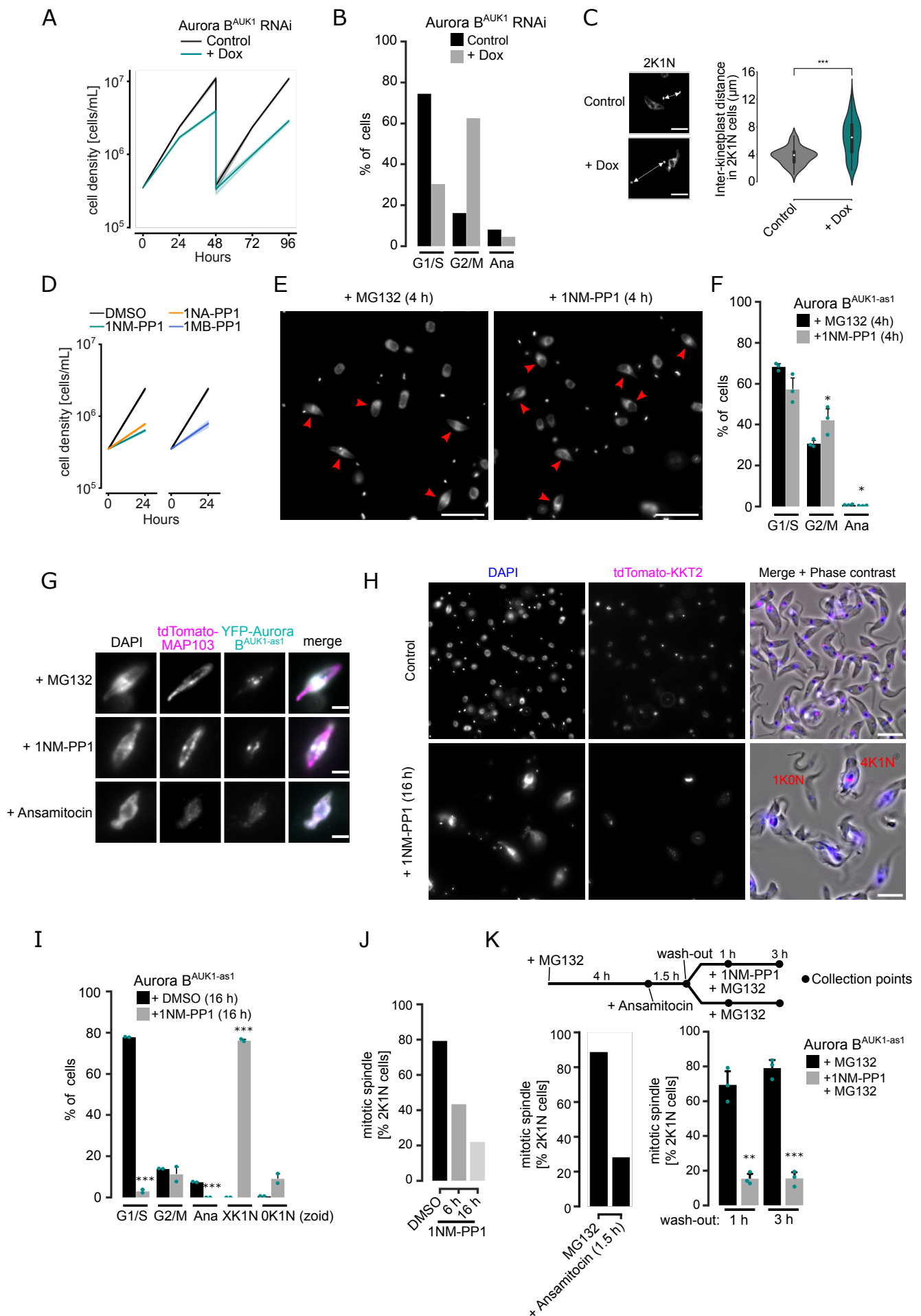

Figure S2

A

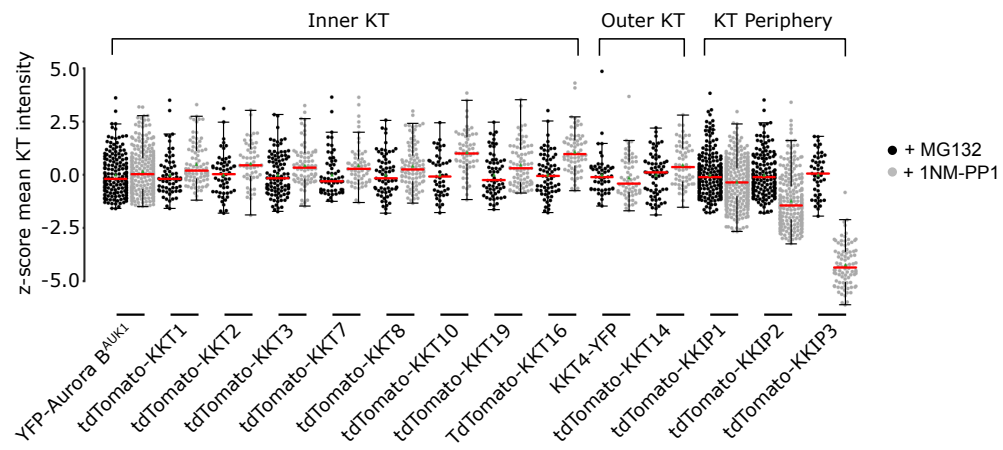

B

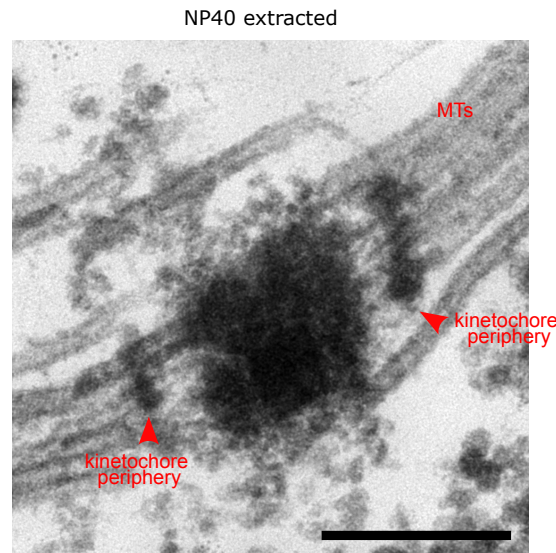

Figure S3

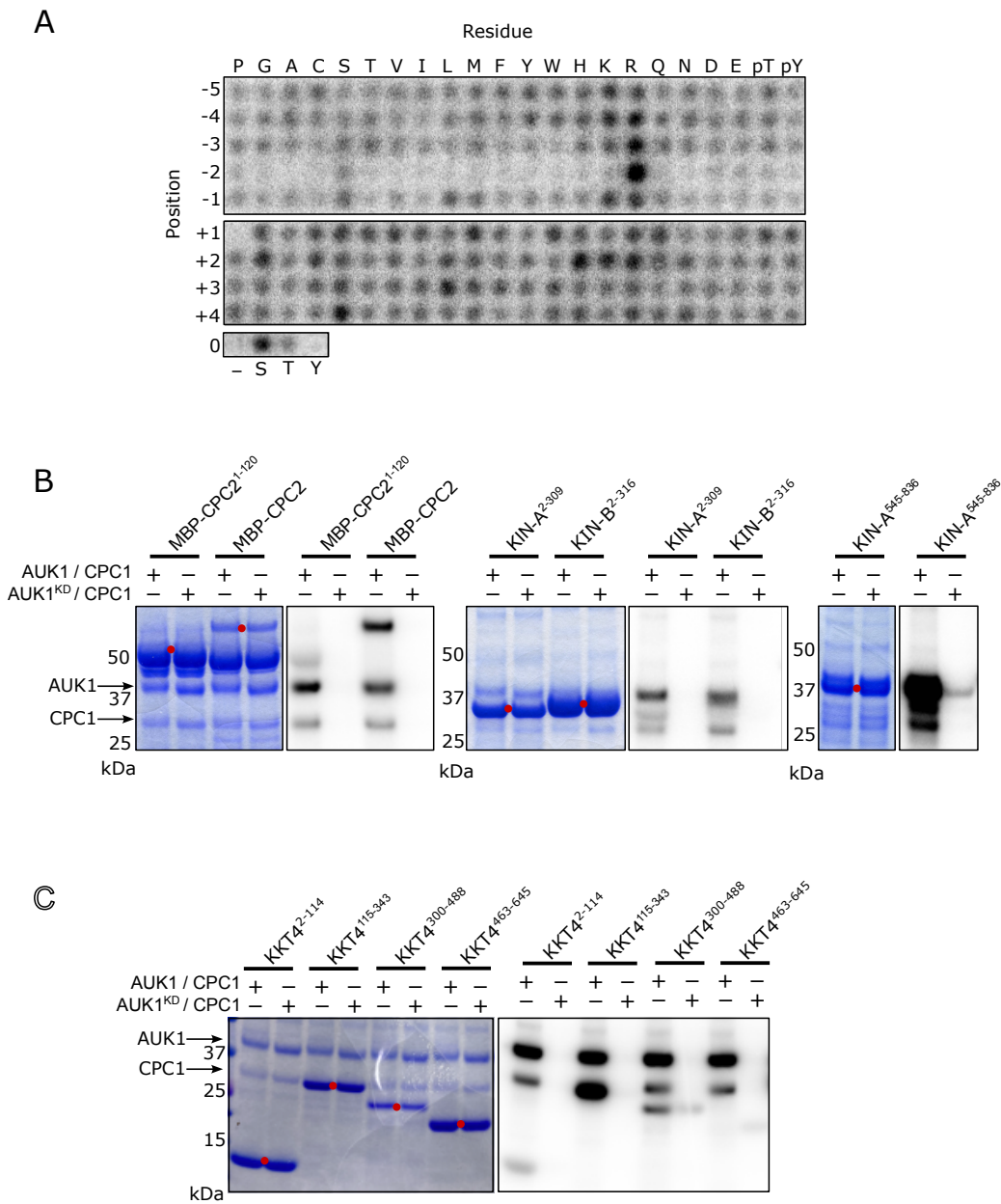

Figure S4

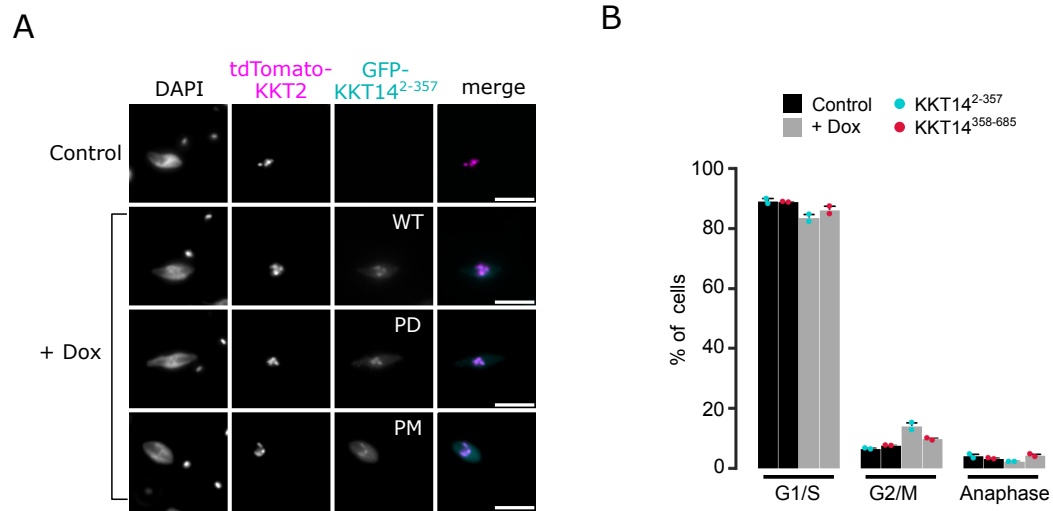

Supplement: Supplement 5 — Figure S1. Aurora BAUK1 activity is required for mitotic exit and spindle stability. (A) Growth curves upon RNAi-mediated knockdown of Aurora BAUK1. RNAi was induced with 1 μg/mL doxycycline and cultures were diluted at day 2. Data are presented as the mean ± SD of three replicates. Cell line: BAP941. (B) Cell cycle profile upon knockdown of Aurora BAUK1. RNAi was induced with 1 μg/mL doxycycline and cells were fixed at 16 h. A minimum of 350 cells per condition were quantified. (C) Quantification of the distance between kinetoplasts in 2K1N cells upon depletion of Aurora BAUK1 for 16 h. A minimum of 50 cells per condition were quantified. Cell line: BAP2129. * P ≤ 0.05, ** P ≤ 0.01, *** P < 0.001 (Mann-Whitney U). (D) Growth curves upon treatment of Aurora BAUK1-as1 cells with 2 μM 1NM-PP1, 1NA-PP1 or 1MB-PP1. Data are presented as the mean ± SD of three replicates. Cell line: BAP2198. (E) Representative fluorescence micrographs showing cell cycle distribution upon treatment of Aurora BAUK1-as1 cells with 10 μM MG132 or 2 μM 1NM-PP1 for 4 h. DNA was stained with DAPI. Red arrowheads indicate 2K1N cells. Cell line: BAP2357. Scale bars, 10 μm. (F) Cell cycle profile for indicated conditions as in (E). All graphs depict the means (bar) ± SD of three replicates (dots). A minimum of 500 cells per replicate were quantified. * P < 0.05, ** P ≤ 0.01, *** P ≤ 0.001 (two-sided, unpaired t-test). (G) Representative fluorescence micrographs showing the localization of the spindle marker tdTomato-MAP103 and YFP-Aurora BAUK1 upon treatment of Aurora BAUK1-as1 cells with 10 μM MG132, 2 μM 1NM-PP1 or 5 nM ansamitocin for 4 h. Cell line: BAP2281. Scale bars, 2 μm. (H) Representative fluorescence micrographs showing an overview of Aurora BAUK1-as1 cells treated with DMSO (Control) or 2 μM 1NM-PP1 for 16 h. Examples of a 1K0N (zoid) and a 4K1N cell are labelled in red. Cell lines: BAP2924. Scale bars, 10 μm. (I) Cell cycle profile for indicated conditions as in (H). All graphs depict [file NIHPP2024.01.20.576407v1-supplement-5.pdf]
